# Supplementary material for: Assessment of Developmental Prosopagnosia in an Individual with Tourette Syndrome and Attention Deficit Hyperactivity Disorder: A Case Report
Source: Brain Sci. 2025 Jan 10;15(1):56. doi: 10.3390/brainsci15010056 (PMC11764325; doi:10.3390/brainsci15010056)
Supplement: Supplementary file 1 [file brainsci-15-00056-s001.zip › brainsci-3392656-supplementary.pdf]

Supplementary material

Table S 1. Primary scores on measurement tools that were not included in the main article.

**Measure**	**Raw score**
Car expertise [1]:	
Objective test of pre-experimental car expertise	9 (41 %)
Level of interest in cars (1 = “no interest at all” and 9 = “extremely interested”)	4
Knowledge of car makes and models from approximately 1985-2005 (1 = “no knowledge at all” and 9 = “extremely knowledgeable”)	3
Reading the Mind in the Eyes test [2]	19
Yale Global Tic Severity Scale - Total Tic Severity Score [3]	26
ADHD symptom severity	
Adult ADHD Self-Report Scale [4]	56
Wender Utah Rating Scale – 25 [5]	52

Note. ADHD = attention deficit hyperactivity disorder.

The car expertise variables (table S 1) allow for the statistical correction for the effect of car expertise on the Cambridge Car Memory Test [1] score. Since we used Danish norms [2] that do not include these covariates, the car expertise scores were not included in the main article. The Reading the Mind in the Eyes test [3] score was not included due to questionable psychometric properties [4]. The Tourette syndrome and attention deficit hyperactivity disorder symptom severity scores [5-7] may be of interest to some readers but were not considered essential to the manuscript.

References

1. Dennett, H.W.; McKone, E.; Tavashmi, R.; Hall, A.; Pidcock, M.; Edwards, M.; Duchaine, B. The Cambridge Car Memory Test: a task matched in format to the Cambridge Face Memory Test, with norms, reliability, sex differences, dissociations from face memory, and expertise effects. *Behavior research methods* **2012**, *44*, 587-605, doi:10.3758/s13428-011-0160-2.
2. Gerlach, C.; Barton, J.J.S.; Albonico, A.; Malaspina, M.; Starrfelt, R. Contrasting domain-general and domain-specific accounts in cognitive neuropsychology: An outline of a new approach with developmental prosopagnosia as a case. *Behav Res Methods* **2022**, *54*, 2829-2842, doi:10.3758/s13428-021-01774-4.
3. Baron-Cohen, S.; Wheelwright, S.; Hill, J.; Raste, Y.; Plumb, I. The "Reading the Mind in the Eyes" Test revised version: a study with normal adults, and adults with Asperger syndrome or high-functioning autism. *J Child Psychol Psychiatry* **2001**, *42*, 241-251.
4. Higgins, W.C.; Ross, R.M.; Langdon, R.; Polito, V. The "Reading the Mind in the Eyes" Test Shows Poor Psychometric Properties in a Large, Demographically Representative U.S. Sample. *Assessment* **2023**, *30*, 1777-1789, doi:10.1177/10731911221124342.
5. Leckman, J.F.; Riddle, M.A.; Hardin, M.T.; Ort, S.I.; Swartz, K.L.; Stevenson, J.; Cohen, D.J. The Yale Global Tic Severity Scale: initial testing of a clinician-rated scale of tic severity. *Journal of the American Academy of Child and Adolescent Psychiatry* **1989**, *28*, 566-573, doi:10.1097/00004583-198907000-00015.
6. Kessler, R.C.; Adler, L.; Ames, M.; Demler, O.; Faraone, S.; Hiripi, E.; Howes, M.J.; Jin, R.; Secnik, K.; Spencer, T.; et al. The World Health Organization Adult ADHD Self-Report Scale (ASRS): a short screening scale for use in the general population. *Psychol Med* **2005**, *35*, 245-256, doi:10.1017/s0033291704002892.
7. Ward, M.F.; Wender, P.H.; Reimherr, F.W. The Wender Utah Rating Scale: an aid in the retrospective diagnosis of childhood attention deficit hyperactivity disorder. *The American journal of psychiatry* **1993**, *150*, 885-890, doi:10.1176/ajp.150.6.885.
